# Tetra­kis(3,5-dimethyl-1*H*-pyrazole-κ*N* ^2^)(nitrato-κ^2^ *O*,*O*′)cadmium(II) nitrate

**DOI:** 10.1107/S160053680803626X

**Published:** 2008-11-13

**Authors:** Su-Qing Wang, Fang-Fang Jian

**Affiliations:** a Microscale Science Institute, Department of Chemistry and Chemical Engineering, Weifang University, Weifang 261061, People’s Republic of China; b Microscale Science Institute, Weifang University, Weifang 261061, People’s Republic of China

## Abstract

The title compound, [Cd(NO_3_)(C_5_H_8_N_2_)_4_]NO_3_, was prepared by reaction of cadmium nitrate and 3,5-dimethyl­pyrazole in ethanol solution. The Cd atom adopts a distorted *cis*-CdO_2_N_4_ octa­hedral geometry involving four dimethylpyrazole molecules and one bidentate nitrate anion. The mol­ecular structure and packing are stabilized by N—H⋯O and C—H⋯O inter- and intra­molecular hydrogen-bonding inter­actions.

## Related literature

For background on the coordination chemistry of Cd(II) in biological systems, see: Dressing *et al.* (1982 ▶). For related literature, see: Addison *et al.* (1984 ▶).
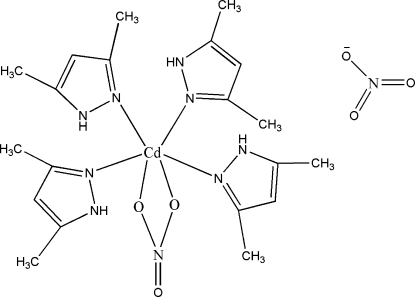


## Experimental

### 

#### Crystal data

[Cd(NO_3_)(C_5_H_8_N_2_)_4_]NO_3_
*M* *_r_* = 620.97
Triclinic, 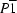

*a* = 9.1790 (18) Å
*b* = 11.353 (2) Å
*c* = 13.669 (3) Å
α = 94.79 (3)°
β = 105.61 (3)°
γ = 90.68 (3)°
*V* = 1366.2 (5) Å^3^
*Z* = 2
Mo *K*α radiation
μ = 0.85 mm^−1^
*T* = 293 (2) K
0.25 × 0.20 × 0.18 mm

#### Data collection

Bruker SMART CCD area-detector diffractometer
Absorption correction: none
7482 measured reflections
5035 independent reflections
4677 reflections with *I* > 2σ(*I*)
*R*_int_ = 0.016

#### Refinement

*R*[*F* ^2^ > 2σ(*F* ^2^)] = 0.046
*wR*(*F* ^2^) = 0.137
*S* = 1.06
5035 reflections
319 parameters
1 restraint
H-atom parameters constrained
Δρ_max_ = 1.66 e Å^−3^
Δρ_min_ = −0.87 e Å^−3^

### 

Data collection: *SMART* (Bruker, 1997 ▶); cell refinement: *SAINT* (Bruker, 1997 ▶); data reduction: *SAINT*; program(s) used to solve structure: *SHELXS97* (Sheldrick, 2008 ▶); program(s) used to refine structure: *SHELXL97* (Sheldrick, 2008 ▶); molecular graphics: *SHELXTL* (Sheldrick, 2008 ▶); software used to prepare material for publication: *SHELXTL*.

## Supplementary Material

Crystal structure: contains datablocks global, I. DOI: 10.1107/S160053680803626X/at2668sup1.cif

Structure factors: contains datablocks I. DOI: 10.1107/S160053680803626X/at2668Isup2.hkl

Additional supplementary materials:  crystallographic information; 3D view; checkCIF report

## Figures and Tables

**Table 1:** Hydrogen-bond geometry (Å, °)

*D*—H⋯*A*	*D*—H	H⋯*A*	*D*⋯*A*	*D*—H⋯*A*
N3—H4*A*⋯O1^i^	0.86	2.13	2.943 (8)	157
N3—H4*A*⋯O3^i^	0.86	2.50	3.289 (16)	152
N4—H5*A*⋯O1^i^	0.86	1.92	2.771 (8)	171
N6—H7*A*⋯O6^ii^	0.86	2.33	3.131 (6)	156
N9—H10*A*⋯O4	0.86	2.50	3.106 (5)	128
C5—H5*B*⋯O4	0.96	2.59	3.530 (7)	166
C10—H10*B*⋯O5	0.96	2.29	3.157 (7)	150
C13—H13*A*⋯O1^iii^	0.93	2.49	3.375 (8)	159

Symmetry codes: (i) 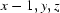; (ii) 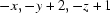; (iii) 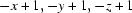.

**Fig. 1.**
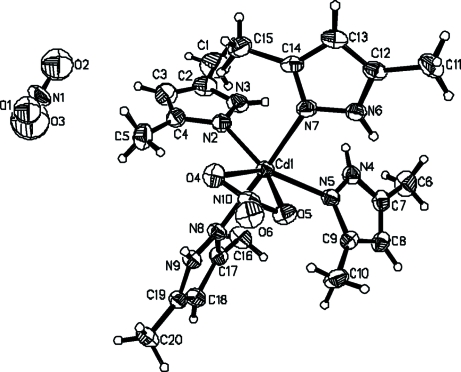
The molecular structure and atom-labeling scheme for (I), with displacement ellipsoids drawn at the 30% probability level.

## supplementary crystallographic information

### Comment

It is also known that most of the Cd(II) in biological systems is not in the form of free Cd(II) ions, but is coordinated by the abundance of biological ligands (Dressing *et al.*, 1982). Therefore the coordination chemistry of Cd(II) with ligands is of great interest. In this paper, we reported the synthesis and the crystal structure of tri(3,5-dimenthyl pyrazolyl)cadmium(II) nitrate (I).

In the molecule of (I) (Fig. 1), each Cd atoms is coordinated by four nitrogen atoms from four 3,5-dimethyl pyrazoles respectively and two oxygen atoms from nitrate anion. All the bond length and angle are in the normal range. Another nitrate anion exists in the crystal lattice.

The molecular structure and packing are stabilized by the N—H···O and C—H···O inter and intraintermolecular hydrogen-bonding intercations.

### Experimental

Solid 3,5-dimethyl pyrazole 0.96 g (0.01 mol) and cadmium nitrate 0.77 g (0.0025 mol) were added in 50 ml anhydrous alcohol under stirring. The mixture was refluxed for 3.5 h. The colourless solution was filtered and the filtrate was left to stand undisturbed. Upon slow evaporation at room temperature, a colourless crystalline solid appeared three days later and was separated by filtration.

### Refinement

H atoms were positioned geometrically and allowed to ride on their parent atoms, with C—H distances of 0.93 and 0.96 Å, and with *U*_iso_(H) = 1.2 or 1.5*U*_eq_ of the parent atoms.

### Crystal data

**Table:**

[Cd(NO_3_)(C_5_H_8_N_2_)_4_]N_1_O_3_	*Z* = 2
*M**_r_* = 620.97	*F*_000_ = 636
Triclinic, *P*1	*D*_x_ = 1.509 Mg m^−^^3^
Hall symbol: -P 1	Mo *K*α radiation λ = 0.71073 Å
*a* = 9.1790 (18) Å	Cell parameters from 6067 reflections
*b* = 11.353 (2) Å	θ = 2.3–28.2º
*c* = 13.669 (3) Å	µ = 0.85 mm^−^^1^
α = 94.79 (3)º	*T* = 293 (2) K
β = 105.61 (3)º	Block, colourless
γ = 90.68 (3)º	0.25 × 0.20 × 0.18 mm
*V* = 1366.2 (5) Å^3^	

### Data collection

**Table:**

Bruker SMART CCD area-detector diffractometer	4677 reflections with *I* > 2σ(*I*)
Radiation source: fine-focus sealed tube	*R*_int_ = 0.016
Monochromator: graphite	θ_max_ = 25.5º
*T* = 273(2) K	θ_min_ = 2.3º
φ and ω scans	*h* = −10→11
Absorption correction: none	*k* = −13→13
7482 measured reflections	*l* = −16→11
5035 independent reflections	

### Refinement

**Table:**

Refinement on *F*^2^	Secondary atom site location: difference Fourier map
Least-squares matrix: full	Hydrogen site location: inferred from neighbouring sites
*R*[*F*^2^ > 2σ(*F*^2^)] = 0.047	H-atom parameters constrained
*wR*(*F*^2^) = 0.137	*w* = 1/[σ^2^(*F*_o_^2^) + (0.0885*P*)^2^ + 1.8569*P*] where *P* = (*F*_o_^2^ + 2*F*_c_^2^)/3
*S* = 1.06	(Δ/σ)_max_ < 0.001
5035 reflections	Δρ_max_ = 1.66 e Å^−^^3^
319 parameters	Δρ_min_ = −0.87 e Å^−^^3^
1 restraint	Extinction correction: none
Primary atom site location: structure-invariant direct methods	

### Special details

**Table:**

Geometry. All e.s.d.'s (except the e.s.d. in the dihedral angle between two l.s. planes) are estimated using the full covariance matrix. The cell e.s.d.'s are taken into account individually in the estimation of e.s.d.'s in distances, angles and torsion angles; correlations between e.s.d.'s in cell parameters are only used when they are defined by crystal symmetry. An approximate (isotropic) treatment of cell e.s.d.'s is used for estimating e.s.d.'s involving l.s. planes.
Refinement. Refinement of *F*^2^ against ALL reflections. The weighted *R*-factor *wR* and goodness of fit *S* are based on *F*^2^, conventional *R*-factors *R* are based on *F*, with *F* set to zero for negative *F*^2^. The threshold expression of *F*^2^ > σ(*F*^2^) is used only for calculating *R*-factors(gt) *etc*. and is not relevant to the choice of reflections for refinement. *R*-factors based on *F*^2^ are statistically about twice as large as those based on *F*, and *R*- factors based on ALL data will be even larger.

### Fractional atomic coordinates and isotropic or equivalent isotropic displacement parameters (Å^2^)

**Table:**

	*x*	*y*	*z*	*U*_iso_*/*U*_eq_	
Cd1	0.13316 (3)	0.80806 (2)	0.33031 (2)	0.03976 (14)	
O4	0.3597 (4)	0.8966 (3)	0.4877 (3)	0.0559 (6)	
O5	0.1497 (4)	0.9850 (3)	0.4459 (3)	0.0538 (6)	
O6	0.3174 (5)	1.0465 (4)	0.5840 (3)	0.0679 (9)	
N2	0.2219 (5)	0.6381 (3)	0.2677 (3)	0.0492 (7)	
N3	0.1348 (5)	0.5493 (4)	0.2059 (4)	0.0600 (9)	
H4A	0.0377	0.5444	0.1918	0.072*	
N4	−0.2135 (4)	0.7666 (4)	0.1863 (3)	0.0497 (7)	
H5A	−0.2040	0.6919	0.1909	0.060*	
N5	−0.1073 (4)	0.8506 (3)	0.2393 (3)	0.0451 (6)	
N6	−0.0798 (5)	0.7578 (3)	0.4743 (3)	0.0491 (7)	
H7A	−0.1426	0.8045	0.4397	0.059*	
N7	0.0527 (5)	0.7270 (3)	0.4544 (3)	0.0495 (6)	
N8	0.2600 (4)	0.9168 (3)	0.2410 (3)	0.0461 (6)	
N9	0.3644 (4)	1.0038 (3)	0.2868 (3)	0.0448 (7)	
H10A	0.4081	1.0120	0.3512	0.054*	
N10	0.2779 (4)	0.9758 (3)	0.5082 (3)	0.0464 (7)	
C1	0.1461 (12)	0.3654 (6)	0.0977 (7)	0.112 (3)	
H1A	0.2229	0.3187	0.0796	0.167*	
H1B	0.0800	0.3925	0.0373	0.167*	
H1C	0.0887	0.3183	0.1303	0.167*	
C2	0.2191 (8)	0.4702 (5)	0.1697 (5)	0.0705 (13)	
C3	0.3657 (8)	0.5085 (5)	0.2081 (5)	0.0731 (14)	
H3A	0.4503	0.4715	0.1967	0.088*	
C4	0.3647 (6)	0.6135 (4)	0.2678 (4)	0.0526 (9)	
C5	0.4955 (6)	0.6919 (6)	0.3272 (5)	0.0731 (16)	
H5B	0.4594	0.7572	0.3622	0.110*	
H5C	0.5480	0.7210	0.2816	0.110*	
H5D	0.5632	0.6478	0.3760	0.110*	
C6	−0.4658 (7)	0.7409 (7)	0.0621 (6)	0.0848 (19)	
H6A	−0.5406	0.7909	0.0249	0.127*	
H6B	−0.5090	0.6967	0.1050	0.127*	
H6C	−0.4327	0.6872	0.0151	0.127*	
C7	−0.3346 (5)	0.8148 (5)	0.1261 (4)	0.0537 (9)	
C8	−0.3053 (6)	0.9339 (5)	0.1395 (4)	0.0543 (9)	
H8A	−0.3682	0.9912	0.1079	0.065*	
C9	−0.1645 (5)	0.9538 (4)	0.2091 (3)	0.0464 (8)	
C10	−0.0833 (7)	1.0697 (5)	0.2516 (5)	0.0689 (14)	
H10B	0.0128	1.0559	0.2979	0.103*	
H10C	−0.1429	1.1160	0.2873	0.103*	
H10D	−0.0676	1.1116	0.1969	0.103*	
C11	−0.2363 (8)	0.7287 (5)	0.5941 (5)	0.0694 (13)	
H11A	−0.3011	0.7812	0.5520	0.104*	
H11B	−0.2042	0.7641	0.6630	0.104*	
H11C	−0.2906	0.6553	0.5920	0.104*	
C12	−0.1004 (6)	0.7059 (4)	0.5551 (3)	0.0509 (8)	
C13	0.0213 (7)	0.6372 (4)	0.5865 (4)	0.0572 (9)	
H13A	0.0383	0.5892	0.6401	0.069*	
C14	0.1155 (6)	0.6525 (4)	0.5233 (3)	0.0475 (7)	
C15	0.2634 (6)	0.5992 (5)	0.5268 (5)	0.0657 (12)	
H15A	0.3006	0.6251	0.4726	0.099*	
H15B	0.2510	0.5145	0.5189	0.099*	
H15C	0.3345	0.6234	0.5912	0.099*	
C16	0.1073 (7)	0.8553 (6)	0.0647 (4)	0.0666 (14)	
H16A	0.0727	0.7937	0.0979	0.100*	
H16B	0.1547	0.8208	0.0150	0.100*	
H16C	0.0228	0.8998	0.0314	0.100*	
C17	0.2195 (5)	0.9358 (4)	0.1424 (3)	0.0462 (8)	
C18	0.2983 (6)	1.0353 (4)	0.1273 (4)	0.0528 (9)	
H18A	0.2891	1.0679	0.0658	0.063*	
C19	0.3919 (5)	1.0756 (4)	0.2202 (4)	0.0459 (8)	
C20	0.5065 (7)	1.1759 (5)	0.2532 (5)	0.0652 (13)	
H20A	0.5515	1.1781	0.3254	0.098*	
H20B	0.4578	1.2490	0.2373	0.098*	
H20C	0.5835	1.1650	0.2180	0.098*	
N1	0.7643 (6)	0.4506 (5)	0.1574 (5)	0.0806 (16)	
O1	0.8224 (7)	0.5308 (6)	0.2225 (5)	0.1116 (18)*	
O2	0.7028 (11)	0.3695 (9)	0.1665 (7)	0.164 (3)*	
O3	0.7830 (17)	0.4749 (13)	0.0795 (12)	0.247 (6)*	

### Atomic displacement parameters (Å^2^)

**Table:**

	*U*^11^	*U*^22^	*U*^33^	*U*^12^	*U*^13^	*U*^23^
Cd1	0.0405 (2)	0.0371 (2)	0.0402 (2)	−0.00263 (12)	0.00835 (13)	0.00418 (12)
O4	0.0536 (12)	0.0644 (18)	0.0442 (11)	0.0005 (9)	0.0059 (9)	−0.0028 (10)
O5	0.0618 (15)	0.0429 (11)	0.0496 (14)	0.0006 (11)	0.0051 (9)	−0.0030 (9)
O6	0.079 (2)	0.068 (2)	0.0463 (17)	−0.0047 (17)	0.0045 (13)	−0.0119 (13)
N2	0.0505 (12)	0.0401 (10)	0.0565 (19)	−0.0024 (10)	0.0156 (13)	−0.0010 (10)
N3	0.0675 (15)	0.0397 (16)	0.070 (3)	−0.0119 (14)	0.0175 (19)	−0.0051 (14)
N4	0.0429 (13)	0.0510 (12)	0.051 (2)	−0.0062 (11)	0.0065 (11)	0.0048 (14)
N5	0.0432 (9)	0.0431 (12)	0.0471 (15)	−0.0030 (8)	0.0076 (8)	0.0091 (11)
N6	0.0595 (18)	0.0430 (19)	0.0497 (19)	0.0036 (14)	0.0214 (15)	0.0103 (13)
N7	0.0581 (15)	0.0460 (15)	0.0482 (16)	0.0079 (11)	0.0175 (12)	0.0144 (12)
N8	0.0434 (14)	0.0460 (14)	0.0456 (10)	−0.0075 (11)	0.0050 (10)	0.0097 (11)
N9	0.0458 (18)	0.0451 (18)	0.0430 (12)	−0.0069 (11)	0.0113 (12)	0.0045 (12)
N10	0.0557 (16)	0.0430 (17)	0.0392 (15)	−0.0088 (11)	0.0116 (10)	0.0023 (9)
C1	0.148 (6)	0.059 (4)	0.116 (6)	−0.019 (4)	0.031 (5)	−0.038 (3)
C2	0.093 (2)	0.044 (2)	0.072 (3)	−0.0035 (19)	0.022 (3)	−0.0101 (18)
C3	0.0842 (19)	0.053 (3)	0.088 (4)	0.004 (2)	0.040 (3)	−0.012 (2)
C4	0.0555 (13)	0.045 (2)	0.059 (3)	0.0029 (12)	0.0205 (19)	0.0004 (15)
C5	0.0479 (17)	0.077 (3)	0.086 (4)	0.002 (2)	0.010 (3)	−0.013 (3)
C6	0.054 (3)	0.100 (3)	0.082 (4)	−0.009 (2)	−0.010 (2)	−0.004 (3)
C7	0.0426 (17)	0.0707 (17)	0.045 (2)	0.0005 (13)	0.0080 (12)	0.0024 (18)
C8	0.0503 (19)	0.0663 (15)	0.045 (2)	0.0131 (14)	0.0091 (14)	0.0079 (18)
C9	0.0482 (17)	0.0466 (11)	0.045 (2)	0.0081 (10)	0.0124 (13)	0.0054 (15)
C10	0.071 (3)	0.0418 (14)	0.085 (4)	0.0075 (18)	0.006 (3)	0.005 (2)
C11	0.089 (3)	0.066 (3)	0.064 (3)	−0.009 (2)	0.043 (3)	0.000 (2)
C12	0.074 (2)	0.039 (2)	0.043 (2)	−0.0074 (16)	0.0224 (17)	−0.0003 (14)
C13	0.081 (3)	0.046 (2)	0.044 (2)	−0.0043 (18)	0.0141 (18)	0.0110 (15)
C14	0.0612 (19)	0.033 (2)	0.044 (2)	−0.0021 (14)	0.0061 (15)	0.0065 (13)
C15	0.061 (2)	0.051 (3)	0.078 (4)	0.0071 (19)	0.003 (2)	0.016 (2)
C16	0.067 (3)	0.080 (3)	0.0456 (19)	−0.015 (2)	0.0061 (19)	−0.002 (2)
C17	0.047 (2)	0.053 (2)	0.0399 (11)	−0.0012 (14)	0.0138 (13)	0.0033 (13)
C18	0.060 (3)	0.057 (2)	0.0459 (14)	−0.0009 (16)	0.0198 (16)	0.0114 (15)
C19	0.047 (2)	0.0426 (19)	0.0525 (16)	−0.0011 (13)	0.0213 (15)	0.0070 (14)
C20	0.071 (3)	0.055 (3)	0.076 (3)	−0.0182 (19)	0.032 (2)	0.003 (2)
N1	0.074 (3)	0.075 (3)	0.085 (4)	−0.042 (3)	0.008 (3)	0.012 (3)

### Geometric parameters (Å, °)

**Table:**

Cd1—N7	2.278 (4)	C6—C7	1.486 (7)
Cd1—N2	2.293 (4)	C6—H6A	0.9600
Cd1—N5	2.303 (4)	C6—H6B	0.9600
Cd1—N8	2.314 (4)	C6—H6C	0.9600
Cd1—O5	2.427 (3)	C7—C8	1.362 (7)
Cd1—O4	2.673 (3)	C8—C9	1.386 (7)
O4—N10	1.240 (5)	C8—H8A	0.9300
O5—N10	1.264 (5)	C9—C10	1.497 (7)
O6—N10	1.225 (5)	C10—H10B	0.9600
N2—C4	1.343 (6)	C10—H10C	0.9600
N2—N3	1.358 (6)	C10—H10D	0.9600
N3—C2	1.341 (8)	C11—C12	1.500 (7)
N3—H4A	0.8600	C11—H11A	0.9600
N4—C7	1.346 (6)	C11—H11B	0.9600
N4—N5	1.365 (5)	C11—H11C	0.9600
N4—H5A	0.8600	C12—C13	1.363 (8)
N5—C9	1.341 (6)	C13—C14	1.396 (7)
N6—C12	1.350 (6)	C13—H13A	0.9300
N6—N7	1.359 (6)	C14—C15	1.483 (7)
N6—H7A	0.8600	C15—H15A	0.9600
N7—C14	1.337 (6)	C15—H15B	0.9600
N8—C17	1.335 (6)	C15—H15C	0.9600
N8—N9	1.356 (5)	C16—C17	1.500 (7)
N9—C19	1.342 (6)	C16—H16A	0.9600
N9—H10A	0.8600	C16—H16B	0.9600
C1—C2	1.505 (8)	C16—H16C	0.9600
C1—H1A	0.9600	C17—C18	1.393 (7)
C1—H1B	0.9600	C18—C19	1.366 (7)
C1—H1C	0.9600	C18—H18A	0.9300
C2—C3	1.357 (9)	C19—C20	1.495 (7)
C3—C4	1.389 (7)	C20—H20A	0.9600
C3—H3A	0.9300	C20—H20B	0.9600
C4—C5	1.490 (7)	C20—H20C	0.9600
C5—H5B	0.9600	N1—O2	1.108 (10)
C5—H5C	0.9600	N1—O3	1.176 (15)
C5—H5D	0.9600	N1—O1	1.226 (8)
			
N7—Cd1—N2	96.81 (14)	C7—C6—H6B	109.5
N7—Cd1—N5	93.80 (14)	H6A—C6—H6B	109.5
N2—Cd1—N5	113.69 (15)	C7—C6—H6C	109.5
N7—Cd1—N8	164.83 (15)	H6A—C6—H6C	109.5
N2—Cd1—N8	89.43 (14)	H6B—C6—H6C	109.5
N5—Cd1—N8	96.33 (13)	N4—C7—C8	105.9 (4)
N7—Cd1—O5	81.49 (13)	N4—C7—C6	121.7 (5)
N2—Cd1—O5	154.81 (14)	C8—C7—C6	132.3 (5)
N5—Cd1—O5	91.50 (13)	C7—C8—C9	107.4 (4)
N8—Cd1—O5	86.98 (13)	C7—C8—H8A	126.3
N7—Cd1—O4	83.20 (13)	C9—C8—H8A	126.3
N2—Cd1—O4	105.38 (13)	N5—C9—C8	109.8 (4)
N5—Cd1—O4	140.88 (12)	N5—C9—C10	122.0 (4)
N8—Cd1—O4	81.82 (12)	C8—C9—C10	128.2 (4)
O5—Cd1—O4	49.43 (12)	C9—C10—H10B	109.5
N10—O4—Cd1	90.4 (2)	C9—C10—H10C	109.5
N10—O5—Cd1	101.7 (3)	H10B—C10—H10C	109.5
C4—N2—N3	105.2 (4)	C9—C10—H10D	109.5
C4—N2—Cd1	128.6 (3)	H10B—C10—H10D	109.5
N3—N2—Cd1	125.3 (3)	H10C—C10—H10D	109.5
C2—N3—N2	111.5 (5)	C12—C11—H11A	109.5
C2—N3—H4A	124.2	C12—C11—H11B	109.5
N2—N3—H4A	124.2	H11A—C11—H11B	109.5
C7—N4—N5	112.0 (4)	C12—C11—H11C	109.5
C7—N4—H5A	124.0	H11A—C11—H11C	109.5
N5—N4—H5A	124.0	H11B—C11—H11C	109.5
C9—N5—N4	104.9 (4)	N6—C12—C13	106.1 (4)
C9—N5—Cd1	130.1 (3)	N6—C12—C11	121.7 (5)
N4—N5—Cd1	123.7 (3)	C13—C12—C11	132.2 (5)
C12—N6—N7	111.5 (4)	C12—C13—C14	107.2 (4)
C12—N6—H7A	124.2	C12—C13—H13A	126.4
N7—N6—H7A	124.2	C14—C13—H13A	126.4
C14—N7—N6	105.9 (4)	N7—C14—C13	109.2 (4)
C14—N7—Cd1	132.7 (3)	N7—C14—C15	122.2 (5)
N6—N7—Cd1	121.3 (3)	C13—C14—C15	128.6 (5)
C17—N8—N9	105.2 (4)	C14—C15—H15A	109.5
C17—N8—Cd1	129.0 (3)	C14—C15—H15B	109.5
N9—N8—Cd1	122.8 (3)	H15A—C15—H15B	109.5
C19—N9—N8	112.3 (4)	C14—C15—H15C	109.5
C19—N9—H10A	123.9	H15A—C15—H15C	109.5
N8—N9—H10A	123.9	H15B—C15—H15C	109.5
O6—N10—O4	123.0 (4)	C17—C16—H16A	109.5
O6—N10—O5	119.2 (4)	C17—C16—H16B	109.5
O4—N10—O5	117.8 (4)	H16A—C16—H16B	109.5
C2—C1—H1A	109.5	C17—C16—H16C	109.5
C2—C1—H1B	109.5	H16A—C16—H16C	109.5
H1A—C1—H1B	109.5	H16B—C16—H16C	109.5
C2—C1—H1C	109.5	N8—C17—C18	109.8 (4)
H1A—C1—H1C	109.5	N8—C17—C16	121.7 (4)
H1B—C1—H1C	109.5	C18—C17—C16	128.5 (4)
N3—C2—C3	106.9 (5)	C19—C18—C17	106.8 (4)
N3—C2—C1	120.8 (6)	C19—C18—H18A	126.6
C3—C2—C1	132.3 (6)	C17—C18—H18A	126.6
C2—C3—C4	106.6 (5)	N9—C19—C18	105.9 (4)
C2—C3—H3A	126.7	N9—C19—C20	121.6 (5)
C4—C3—H3A	126.7	C18—C19—C20	132.5 (5)
N2—C4—C3	109.8 (5)	C19—C20—H20A	109.5
N2—C4—C5	121.6 (4)	C19—C20—H20B	109.5
C3—C4—C5	128.7 (5)	H20A—C20—H20B	109.5
C4—C5—H5B	109.5	C19—C20—H20C	109.5
C4—C5—H5C	109.5	H20A—C20—H20C	109.5
H5B—C5—H5C	109.5	H20B—C20—H20C	109.5
C4—C5—H5D	109.5	O2—N1—O3	124.1 (11)
H5B—C5—H5D	109.5	O2—N1—O1	128.2 (8)
H5C—C5—H5D	109.5	O3—N1—O1	107.6 (9)
C7—C6—H6A	109.5		

### Hydrogen-bond geometry (Å, °)

**Table:**

*D*—H···*A*	*D*—H	H···*A*	*D*···*A*	*D*—H···*A*
N3—H4A···O1^i^	0.86	2.13	2.943 (8)	157
N3—H4A···O3^i^	0.86	2.50	3.289 (16)	152
N4—H5A···O1^i^	0.86	1.92	2.771 (8)	171
N6—H7A···O6^ii^	0.86	2.33	3.131 (6)	156
N9—H10A···O4	0.86	2.50	3.106 (5)	128
C5—H5B···O4	0.96	2.59	3.530 (7)	166
C10—H10B···O5	0.96	2.29	3.157 (7)	150
C13—H13A···O1^iii^	0.93	2.49	3.375 (8)	159

Symmetry codes: (i) *x*−1, *y*, *z*; (ii) −*x*, −*y*+2, −*z*+1; (iii) −*x*+1, −*y*+1, −*z*+1.
